# Community participation for malaria elimination in tafea province, vanuatu: part ii. social and cultural aspects of treatment-seeking behaviour

**DOI:** 10.1186/1475-2875-10-204

**Published:** 2011-07-26

**Authors:** Anna Tynan, Jo-An Atkinson, Hilson Toaliu, George Taleo, Lisa Fitzgerald, Maxine Whittaker, Ian Riley, Mark Schubert, Andrew Vallely

**Affiliations:** 1 Pacific Malaria Initiative Support Centre, School of Population Health, University of Queensland, (Herston Road), Brisbane, (4006), Australia; 2 Public Health Interventions Research Group, The Kirby Institute, University of New South Wales, (West Street) Sydney, (2010), Australia; 3 Save the Children, Port Vila, Vanuatu; 4 National Vector Borne Disease Control Programme, Ministry of Health, Port Vila, Vanuatu; 5 Australian Centre for International and Tropical Health, School of Population Health, University of Queensland, (Herston Road), Brisbane, (4006), Australia

## Abstract

**Background:**

Early diagnosis and prompt effective case management are important components of any malaria elimination strategy. Tafea Province, Vanuatu has a rich history of traditional practices and beliefs, which have been integrated with missionary efforts and the introduction of modern constructions of health. Gaining a detailed knowledge of community perceptions of malarial symptomatology and treatment-seeking behaviours is essential in guiding effective community participation strategies for malaria control and elimination.

**Method:**

An ethnographic study involving nine focus group discussions (FGD), 12 key informant interviews (KII) and seven participatory workshops were carried out on Tanna Island, Vanuatu. Villages in areas of high and low malaria transmission risk were selected. Four ni-Vanuatu research officers, including two from Tanna, were trained and employed to conduct the research. Data underwent thematic analysis to examine treatment-seeking behaviour and community perceptions of malaria.

**Results:**

Malaria was perceived to be a serious, but relatively new condition, and in most communities, identified as being apparent only after independence in 1980. Severe fever in the presence of other key symptoms triggered a diagnosis of malaria by individuals. Use of traditional or home practices was common: perceived vulnerability of patient and previous experience with malaria impacted on the time taken to seek treatment at a health facility. Barriers to health care access and reasons for delay in care-seeking included the availability of health worker and poor community infrastructure.

**Conclusion:**

Due to programme success of achieving low malaria transmission, Tafea province has been identified for elimination of malaria by 2012 in the Government of Vanuatu Malaria Action Plans (MAP). An effective malaria elimination programme requires interactions between the community and its leaders, malaria workers and health providers for success in diagnosis and prompt treatment. As malaria becomes more uncommon, utilizing unique motivators for communities to seek early diagnosis and treatment is important, particularly as other health conditions that cause fevers become increasingly more common. The design of these interventions are dependent upon robust understanding of community perceptions of disease, and the evolving nature of these perceptions.

## Background

Early diagnosis and effective treatment of all malaria cases is an essential component of an elimination campaign [1]. This not only requires appropriate infrastructure and resourcing at primary health care facilities, but also active engagement and participation of communities to recognize malaria symptoms and access treatment at formal health facilities in order to reduce the reservoir of malaria parasites [1]. Tafea Province, Vanuatu, has been targeted for elimination of malaria by 2014 in the Government of Vanuatu Malaria Action Plan (MAP). Support for the MAP is provided through the National Vector Borne Disease Control Programme (NVBDCP) and Ministry of Health (MoH), with assistance from 'The Global Fund' to fight AIDS, Tuberculosis and Malaria, World Health Organization (WHO) and the Australian Government's AusAID Pacific Malaria Initiative (PacMISC) [2]. The focus of this paper is to explore the community perspectives of malaria within Tannese communities and how local constructions of health and illness impact on treatment-seeking behaviour for malaria. This investigation is part of a larger research project examining community perceptions on Tanna Island, Vanuatu of prevention and treatment-seeking behavior for malaria, in order to identify key strategies for sustained community participation for malaria elimination in the context of a low transmission setting [3]. An analysis of health priorities and acceptability of prevention practices has been described previously [3]. This work was conducted at the request of the Government of Vanuatu in order to inform community engagement and health promotion activities to be undertaken by NVBDCP and the MoH.

Challenges to the elimination of malaria in low transmission settings, such as Tanna Island, include the inability to sustain control programs due to community perceptions and practice for malaria prevention and physical and socio-cultural factors that may impact upon treatment-seeking behavior [4]. The consequence of not sustaining elimination is the risk of resurgence of more severe malaria as a result of lowering naturally acquired partial immunity to the disease [5]. Previous studies from Africa and South East Asia show treatment-seeking behaviour for malaria relies on a number of factors including gender; past experience of the disease, health services and treatments; knowledge from others; familiarity with the term malaria and other introduced knowledge [6-8]. These factors in turn are affected by health service determinants, such as physical access to facilities, cost of and levels of satisfaction with health centre services; beliefs in, acceptability of and satisfaction with traditional medicines; availability of other treatments at home such as left-over medications; and indigenous interpretations of fever and other malarial symptoms [9,10].

The success of the global smallpox eradication campaign was dependent on the availability of a vaccine and a successful campaign of surveillance and containment in the context of favorable epidemiological characteristic of the virus, notably a low level of infectiousness [11]. Surveillance consisted of case-finding through systematic searches, improved reporting systems, and active source tracing [11]. This also required a high level of community engagement including identification of smallpox cases and support of affected people to report promptly and appropriately seek treatment, to maintain and sustain long term community surveillance [11].

Malaria, with no vaccine and relatively less distinct symptomatology compared to smallpox presents a more complex challenge for elimination. Early case detection and treatment relies heavily on an individual's or their caretaker's ability to identify symptoms and access appropriate treatment in a timely manner. Correct diagnosis and access to treatment is important not only for clinical reasons but also for public health purposes to enable the effective design, implementation and evaluation of effective health interventions [12]. Community engagement in active case detection (ACD) for malaria, including accessing appropriate treatment and maintenance of preventative activities, such as utilization of mosquito nets, management of vector breeding sites and continued epidemiological surveillance, is also complex and requires long-term commitment and active participation from communities to prevent malaria re-introduction following successful elimination [13].

Identification of malaria cases by health staff and community members can be difficult and highly variable due to the absence of distinct symptoms and multiple differential diagnosis possibilities [14,15]. This, combined with variations in cultural perceptions and interpretations of symptoms, (which may also be held by community-based health staff), have resulted in health promotion for malaria usually focusing on encouraging early presentation for all fever episodes [16]. Diagnosis of malaria-like illnesses has been described in many societies to be subsumed either under one large illness category or several terms based on the different manifestations of the illness [17-19]. Vanuatu, with a rich Melanesian history coupled with the archetypal advent of traders, missionaries and colonizers, has its own unique and varied traditions of health and illness interpretation [20-23]. These traditions are also prominent within communities of Tanna Island, Tafea province.

Malaria has played a major part in Vanuatu's history and likely influenced population distribution [12,24]. In the late 19^th ^century numerous reports of malarial fever and deaths from missionaries in Vanuatu began to emerge, including a number from Tanna Island [25]. John Paton, a Presbyterian missionary in Kwamera and Port Resolution South Tanna, from 1858 until 1862, wrote extensively in his autobiography of his struggles with "*fever and ague*" (the colonial description of malaria), including the death of his wife and child at Port Resolution from the illness [25]. As Paton described:

*'Unfortunately we learned, when too late, that both houses were too near the shore, exposed to unwholesome miasma, and productive of the dreaded fever and ague, the most virulent and insidious enemy to all Europeans in those Southern Seas.' *[25]

Perceptions about disease aetiology and appropriate treatment are well grounded in the culture, history and traditions of most people [4,14]. On Tanna, as is the case in many traditional societies of the Pacific Islands, causal events are given a spiritual dimension, such as the breaking of taboos, links to internal emotions, sorcery, spiritual figures and "garden magic" (Traditional belief that rituals of growing crops, types of plants and other objects within small family farms have a link to determining health, illness and misfortune of people, families and communities) [26]. These have all been identified as underpinning health, illness and misfortune [12,26]. These customary beliefs have been intertwined with Christian belief systems in spite of missionary efforts to encourage movement away from beliefs based on *Kastom*, the Bislama word used to refer to traditional culture, including religion, economics, art and magic in Melanesia [27].

On Tanna, people draw a distinction between what they define to be traditional knowledge, practice and object (*kastom*), and what they perceive as foreign or innovative (*nariitoga*, foreign thing: *narumnarime sei ni-pitoga*, things of foreigners) [20,22]. Although the health facilities (hospitals, health centres and aid posts) which exist on nearly every island of Vanuatu are equipped to provide modern Western models of health care, *kastom *medicine still thrives, especially on the more remote islands including Tanna [28]. It has been suggested that modern and *kastom *medicine do not compete with one another; but rather, are used as complementary systems [28]. Traditional healers (also known as '*Clevers'*) combine plant knowledge with knowledge of the supernatural and operate as true health therapists within their communities [23,28].

Determining how best to encourage the community to present for early treatment of fever or to participate in ACD (surveillance activities) requires an understanding of indigenous forms and interpretations of the disease that influences decision making processes for treatment-seeking [14,29]. Tanna Island has a history of entwined biomedical and traditional approaches to health and illness. The impact that this has on early diagnosis and treatment of a target disease - one of the cornerstones of malaria elimination programs - needs further exploration. Particularly, how interpretations of health and illness affects the sequencing of treatment-seeking behaviour within a low transmission setting such as Tanna.

This investigation is Part II of a larger research project examining community perceptions on Tanna Island, Vanuatu of prevention and treatment-seeking behaviour for malaria with community health priorities and acceptability of prevention interventions already described in Part I [3]. The purpose of this study was to explore the social, cultural and spiritual underpinnings of malaria knowledge in Tanna and understand how these beliefs influence treatment-seeking behaviours. Structural barriers to treatment-seeking such as access, cost and availability of health workers and other resources were also explored.

## Methods

### Study area

Situated on the southern end of Vanuatu, Tafea Province lies between 18 - 19°S and 169 - 170°E and is made up of four main islands including Tanna island which has an isolated population of about 28,000 [29-31]. Malaria programme implementation, supervision and reporting has been described as a challenge in all areas of Vanuatu including Tanna Island due to difficult geography [32]. The national language of Vanuatu is Bislama, a pidgin language, however Tanna is home to a further 7 local dialects [20,23]. Additionally, there is a strong linguistic English and French influence because of the establishment of French and English school systems in the colonial condominium period [20,21]. The key Christian religious groups operating on Tanna include Seventh-day Adventists, Assemblies of God, Catholic, Presbyterian, Church of Christ, Bahai, Pentecostals, Four Square Gospel and Neil Thomas Ministry. In addition, *kastom *(followers of traditional customs) is still practiced on some parts of Tanna and the John Frum movement has been identified as a major cargo cult of the island [22]. Some villages contain members of up to eight different ideological affiliations. Further details of employment patterns and political history were described in Part I paper of this series [3].

Vanuatu has 5 major hospitals (3 provincial and 2 major referral hospitals) and 27 health centres that provide integrated care by a nurse practitioner. The health centres are the referral point for the 89 active dispensaries and 180 aid posts. Aid posts, each staffed by a village health worker (VHW) have been established in most villages and are funded by the community, while the Ministry of Health provides basic medicine and training for the staff [33]. Access to primary health care in Vanuatu is limited by distances in remote and rural areas such as on Tanna Island. For such reasons, the VHW or Aid Post act as an integral part of the health care system as the first point of call for primary health care in many communities across Vanuatu [33,34].

Traditional medicine is also part of the cultural heritage of Vanuatu and on Tanna, traditional healers are active and offer prescription and guidance for management of ailments including fevers, pregnancy and injuries [26,28,35]. Traditional healers can play a complementary role with western medicine or first line treatment [28]. For the purpose of this investigation *Kastom *medicine was defined as traditional treatment of ailments that is practiced and prescribed with direct involvement from traditional healers [26,28]. Home remedies, on the other hand, incorporated strategies, not necessarily linked to traditional beliefs, used by families to assist in managing ailment symptoms.

This study was carried out over a six-week period from July to August 2009 in three villages across Tanna; (North Tanna, South Tanna and Middle Bush). Villages were selected in collaboration with key community stakeholders considering variations in malaria transmission risk levels (as defined by the NVBDCP based on recent epidemiological surveys [36]) in order to capture potential differences in community attitudes and perceptions [3]. The coastal villages of North and South Tanna were assessed to constitute high malaria transmission risk areas and Middle Bush, an inland area, was considered to have low malaria transmission risk as identified by a parasitology survey conducted by the NVBPCP and Ministry of Health with support from t PacMISC and WHO [37].

### Study procedures

To analyse the community understanding of malaria, its symptoms, causation and how these factors influence treatment-seeking behaviours, a number of qualitative methods were engaged [38]. These methods included nine focus group discussions (FGDs), seven participatory workshops and 12 key informant interviews (KIIs), as well as structured and unstructured observation and field notes. All discussions and interviews were recorded and preliminary results were discussed with key community stakeholders to ensure validity as fieldwork progressed.

The research involved a collaboration between the Vector Borne Disease Control Programme, PacMISC, and Save the Children Vanuatu (Australia) (SCA). Four field research officers were recruited locally by SCA to assist with these research activities. Training of field research officers in qualitative research methods, data management and study logistics was undertaken over a five day intensive course two weeks prior to the planned research activity by members of the research team, which included an experienced social scientist. The two male field research officers were local to Tanna and could speak *Bislama *as well as some of the local dialects in North Tanna and Middle Bush region. The two female officers were both fluent *Bislama *speakers from other islands of Vanuatu. The primary researcher, from SPH, UQ was not ni-Vanuatu or a *Bislama *speaker but provided technical support and supervision to the local field research officers, and directed FGDs and participatory workshops to gather data on key objectives as indicated.

### Participant recruitment

Village leaders assisted in the selection and recruitment of participants for the FGDs, KIIs and participatory workshops. On arrival at a village, meetings were held at the *nakamal *(village meeting place) with the village leaders and household heads to discuss and plan for the week of research activities in their community as is customary protocol. FGDs and participatory workshops were carried out with primary caregivers, women, men and youth and KIIs involved health workers, chiefs, teachers and other village leaders.

### Data collection

KII, FGDs and participatory workshops were conducted in *Bislama*. In the Middle Bush and South Tanna villages further translation was required by participants who did not speak *Bislama *or a language dialect known by the field research team. This was typically done by other participants in the group. All discussions and interviews were digitally recorded with a voice recorder and transcribed and translated into English by the field research officers. Random samples of these translations were also cross-checked to ensure validation of interpretations. In addition, informal observations were documented during the participatory workshops and FGDs by the field researchers and a field journal kept by the primary researcher during the intensive period of data collection. One FGD was inaudible due to background noise, however, comprehensive notes written by the research team following each FGD allowed information to be retrieved from this lost discussion.

Participatory workshops consisted of activities such as free listing and ranking of health and disease priorities in communities to gain an understanding of illness patterns community's priorities and perceptions [6]. In-depth discussions about reasons for priority ranking of illnesses followed these participatory activities and attempts made to elucidate meanings of fever and malaria related symptoms listed.

### Data analysis

Data was triangulated with the transcriptions of the FGDs and KII, reports from the research officers completed immediately after each activity and other field notes taken by the primary researcher. A thematic analysis of all the information was conducted by the primary author to organize, identify, analyse and report patterns (themes) within data [39]. As the analysis progressed, a list of themes and ideas emerging from the data was developed to provide a basis for the results section. Adjustments to themes and ideas were also made as new information and issues arose during the process [39]. To ensure understanding of participants' realities, experience and meanings, a 'realist method' was used to apply to these areas of consensus and divergence [39].

### Ethical considerations

Ethical approval was granted by the Vanuatu Ethics Committee, Ministry of Health and the Behavioural & Social Sciences Ethical Review Committee, University of Queensland, Australia to carry out this research. Written informed consent was obtained from all individuals participating in interviews, group discussions and participatory workshops. Confidentiality was maintained with use of pseudonyms during the data transcription process. Consideration was given by all researchers to the customs, practices and legal systems on Tanna, potential for language barriers and the provision of a clear understanding of research objectives to all participants involved in this study.

## Results

Participants across each village represented a variety of religious affiliations including those identifying with *kastom *and members from the John Frum movement [22]. Across all study villages, the age range of women was 20 - 60+ years, men 26-57 years and youth 15- 34 years. Youths were typically defined by communities as young, unmarried adults, however some youths were married and based their definition of youth as being eligible and active members of youth church groups. Youth were most likely to have primary or secondary education and the women's groups were more likely to have limited formal schooling. Most married women identified themselves as housewives and the typical occupation of adult males was a subsistent farmer. Families were typically made up of large numbers of up to nine children (Table 1).

**Table 1:** Demographic characteristics of participants of the FGDs and participatory workshops

		Adults		Youth	
		
		Males N = 22	Females N = 46	Males N = 30	Females N = 24
		
Age Range					
	Age in years	26 - 57 yrs	20 - 60+yrs	15 - 34 yrs	16 - 21 yrs
**Education**					
	**Primary**	63%	40%	39%	39%
	
	**Secondary**	14%	17%	61%	61%
	
	**None**	23%	43%		

**Occupation**					
	**Subsistent farmer**	87%	12%	70%	22%
	
	**Fisherman**	5%			
	
	**Pastor**	5%			
	
	**Housewife**		77%		
	
	**Carpenter**	5%			
	
	**Teacher**		12%		
	
	**TAFE Student**			30%	78%

**Number of Children**					
		**North Tanna**	**Middle Bush**	**South Tanna**	
		
	**0 children**	6%	0	0	
	
	**1-3 Children**	35%	41%	28%	
	
	**4-6 Children**	35%	45%	55%	
	
	**7-9 Children**	24%	14%	17%	

### Community perceptions of malaria

Fever, as a general concept and experience had a long held place in the memory of all Tannese. However, the emergence of malaria as a term and disease concept distinguished from other fever entailed some uncertainty for many, as one informant typically explained:

*In the past there is fever but there is no malaria, and we don't know where malaria comes from*. (female participant, South Tanna, FGD)

For many, the initial perception of malaria was that it was a new condition that had only arrived in Vanuatu after independence in 1980. However, based upon further questioning about symptomatology during the interviews and FGDs, the disease that had closest similarities to malaria was identified in *Bislama *by the perceived cause, '*sik blong mosquito*' (sickness belonging to mosquito) or primary symptom '*fiva*.' The linguistic signifier *mosquito *utilized to distinguish malarial fever from other fever is best summarized by the following comment:

*Ah..... There are two types of fever ............ one is cause by mosquito and the other one is passed through air*. (female participant, Middle Bush, FGD).

**Figure 1:**
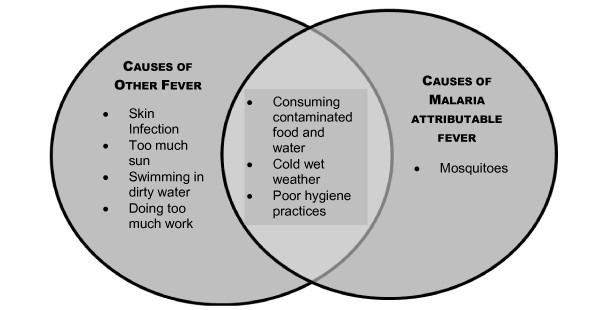
**Differentiating Fever attributed to malaria from other fever by aetiology**.

The perception of causes of malaria or *sik blong mosquito *has previously been described in this research series [3] and recapitulated in Figure 1 which shows the conscious link of malaria with the mosquito by the communities of Tanna. During the research activities, participants were also asked if the community had a local dialect translation for *sik blong mosquito *based on the description of symptomatology that they had come to understand. However, due to difficulties with literal translation, a verified translation could not be found.

### Patterns of differential diagnosis for fever

As the research evolved, it became apparent that most people in Tanna communities tend to identify two types of fever: one that passed *inside *the body and one that passed *outside *the body; a perception described by both youth and adults but particularly older generations. This was best explained by a Middle Bush primary caregiver;

*... when fever is inside means there is hot inside the body and the patient feels like wanting to vomit or diarrhea. That is when the fever is inside. But when it is outside of the skin, is like when the patient sneezes and has running nose, is like cold and flu*. (primary caregiver, Middle Bush, FGD)

The fever caused by *sik blong mosquito *was described as a serious fever and often included a number of additional symptoms including headache, sore body and more closely linked to the description of fever "inside the body." Typically, fever accompanied by a headache and a sore body and joints were seen to be key indicators of *sik blong mosquito*.

For some, duration of the symptoms was important in prompting the person to consider the presence of *sik blong mosquito*. Fever that continues after one day with the symptoms of hot and cold and shivering was described as more likely to be serious, possibly *sik blong mosquito *and in need of treatment. As a community leader in North Tanna explained;

*Sometimes people can get sick from cold and hot then getting better the next day, the following day feeling the same sick then they know that it is fever. If the fever is getting worse then they now know that it is sik blong mosquito, so they have to go to the hospital quickly*. (KII)

During the workshops, participants were asked to list common illnesses within their communities. During this activity other temperature-related symptoms which were different from fever due to *sik blong mosquito *were mentioned but usually only in relation to children. This was common across all villages and included the conditions of '*hot*', '*hot cold*' and '*pikinini i' hot*'(childhood hotness). Hot was described as just feeling hot when *"..the child does not want to eat*." with the possible cause being from "*swimming in dirty water*" according to youth in Middle Bush (Participatory workshop). '*Pikinini i hot' *was noted as caused by being in the sun too long and; hot/cold was used intermittently with (the word) fever to describe the actual occurrence of fever and its characteristics. For one female participant in South Tanna, although not commonly reported, fever was described as a result of *Kastom *dance;

Participant: *... you know when the people part takes in the Kastom dances*

Interviewer: *ooh so they shake their body very hard that they got sick*

Participant: *yes, like they dance very strong that causes body pain that leads to fever. (laughing and giggling)*. (primary caregiver, South Tanna, FGD)

During the FGDs and participatory workshops, fever due to *sik blong mosquito *was distinguished from other fever by a range of symptoms and their characteristics (Table 2). Symptom signifiers were established based on Bislama terms only due to the research team not being fluent in all the local dialects that participants spoke. As noted by many respondents, severity and longevity of the fever are key indicators for *sik blong mosquito*.

**Table 2:** Distinguishing malarial fever from other fever by presentation of symptoms- summary of community responses in Tanna Island, 2009

	**All Other Fever**	**Fever due to cold and flu**	**Fever due to 'sik blong mosquito'**
	
	Fever outside or inside the body	Typically fever inside the body	Fever inside the body
	
	Patient feels hot	Patient feels hot	Patient feels hot and cold*
	
	Loss of appetite	Loss of appetite	Loss of appetite
	
	Dizzy	Dizzy	Dizzy
	
**Symptom Signifiers**	Weak	Weak	Weak
	
	Dehydration possible	Dehydration possible	dehydration
	
	Cough	Cough	Cough
	
	Fever may be only symptom	Fever always with other symptoms	Fever always with other symptoms
	
	Sometimes head ache	Sometimes headache	Always Head ache*
	
	Sometimes body pain	Sometimes body pain	Always Body ache*
	
	Symptoms rarely last for long time	Symptoms sometimes last for long time	Symptoms always lasting 24 hours or more*
	
	Sometimes severe symptoms	Sometime severe symptoms	Always Severely presenting symptoms*

*indicates notable difference in symptom signifiers for malaria.

*Yes, sik blong mosquito is a bit different from fever, because it is urgent and very tough that can spoil people*. (North Tanna, KII).

Fever *due to sik blong mosquito *was also acknowledged as a great concern within all communities visited, with people tending to relate its impact and urgency to death and disability, especially of children.

*...in 1987 a lot of children were sick and needed to go to the hospital for treatment ...the doctor said it was sik blong mosquito and sometimes it caused disability*. (Middle Bush, KII).

Many people were more likely to link their initial awareness of *sik blong mosquito *with personal experience, whether having it themselves or their children having it. Initial awareness of 'sik blong mosquito' was reflected by many participants in graphic descriptions of episodes of particular epidemics. These episodes were usually ones that especially affected children and resulted in complications from severe disease such as disability which included cognitive deficit, hearing and vision impairments. It was also very common for people to describe initial awareness to be from visiting the main island hospital and being diagnosed with *sik blong mosquito*, or from visiting health professionals in the villages.

Despite an obvious concern from community members about the impact of *sik blong mosquito*, distinguishing it from other fevers without the introduction of Western medicine, its concepts and practices has not been fully developed. Such local identification practices still have a great determining influence of treatment-seeking behaviours in Tanna communities.

### Treatment-seeking behaviour for fever

#### Patterns of treatment-seeking behavior

Most people noted that they would not hesitate to go to the aid post if they suspected they had contracted *sik blong mosquito*. However, as already outlined, a number of factors contribute to decision making processes before people consider symptomatology as indicative of fever due to *sik blong mosquito*, or another serious fever. Typical of this was the comment;

*There are two kinds of fever, if it is only fever then you might try home management first, if it is strong fever then people come to the aid post for treatment. Sometimes people can have flu without getting treatment but when it is infection then they must come only to the aid post*. (South Tanna, KII)

**Figure 2:**
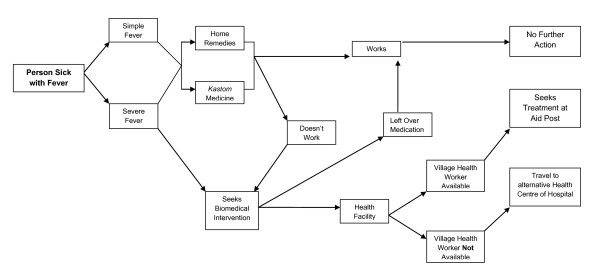
**Hierarchy of resort for treatment seeking behaviour for Fever on Tanna Island**.

There were no differences reported in treatment-seeking behaviour between genders or age groups of the people who participated. When faced with fever people typically responded with a series of protocols (Figure 2). The *kastom *(traditional) medicine or other home remedies were a typical first treatment resort of any type of fever. The methods that are utilized include, washing with special leaves boiled in water, steaming people (covered in blankets with a basin of hot steaming water), drinking prepared leaves or having cold shower or sponge bath.

If symptoms subside after utilizing *kastom *medicine or home remedies, with people waiting up to 24 hours to make a decision, then most reported that they would not proceed to the aid post. However, it was routinely claimed that if the *kastom *medicine or home remedies did not work, respondents would then consider taking the person or themselves to the aid post for further investigation or seeking other forms of biomedical intervention.

It also appeared common for people to share left-over medications from earlier incomplete treatment courses of fever with family and other community members.

*if one of my family members have fever I'll take them to the aid post and take medicine. That person takes that medicine and when she's feeling better than half of the medicine can stay until he/she has fever again but if the treatment doesn't work then I'd take them to the aid post for the health worker to do further treatment*. (primary caregiver, South Tanna, FGD).

Positive and negative previous experience with *sik blong mosquito *treatment also had an impact on treatment-seeking behaviours of some individuals. In one particular example, which illustrates this pattern of treatment resort, a man in South Tanna reported that he went to the aid post, was diagnosed with *sik blong mosquito *and subsequently took the prescribed medication. However, as a result of experiencing the adverse reaction of nausea and vomiting to the prescribed medication, the man resorted to *kastom *medication.

#### Influence on treatment-seeking behaviours

It was common that ideological affiliations influenced the treatment-seeking protocols that people followed for fever:

*...she said she belongs to the Kastom. For them if anyone has fever, they will treat with Kastom medicine first. If the patient still feels sick then that is when they will take to the health centre*. (Primary Caregiver Middle Bush FGD)

For some others, a combination of *kastom *and other religious protocols may be performed. Christian ritual was typically only performed when waiting for further intervention, not as a step in itself.

*... we can take patient to traditional medicine but as Christians we can also offer prayers for healing on the sick patient. We can ask God to heal the patient while trying our best to find a cure for the illness*. (primary caregiver Middle Bush FGD)

In rare instances, people claimed to avoid all *kastom *medicines due to their particular religious views; however, some would still try home remedies such as having a cool bath whilst waiting for biomedical intervention.

Children were considered more susceptible to the impacts of *any *type of fever and seeking care at aid posts and other health centres was often much quicker than for adults (Table 3). Particular attention was also given to pregnant women with many indicating they would seek health centre advice quickly.

**Table 3:** Time to seek treatment from Aid Post following recognized onset of fever

Infant	First 12 hours
Child (5 - 12)	First 24 hours

Adult	Over 24 hours

Pregnant woman	First 24 hours

*We have had one experience like that with a mother when she had sik blong mosquito then she gives birth to her child but then her child died*. (primary caregiver, North Tanna, FGD)

Caution during pregnancy was also noted by almost all women in using any leftover medication due to concerns about the risks of Western medicines in pregnancy and many therefore said they would seek advice at the aid post more readily. Delay in accessing an aide post of other health centre would be more likely if a non-pregnant adult had access to left over medication. Delay in treatment from the aid post was also more common in non-pregnant adults due to being considered stronger and more resilient (Table 3). Severe symptoms in any person old or young would motivate some households to seek immediate attention and access to the aid post or hospital.

#### Barriers to treatment-seeking behaviour

Common factors influencing decisions to access formal healthcare services were: perceptions of fever causation and severity, and availability of alternatives (that may include the use of home remedies, *kastom *medicine or left over medication). A number of other circumstances leading to delays in being diagnosed and treated were identified. These included waiting time at the health facility, opening hours and availability of treatment, summarized typically by the following quote;

*The patient may have to wait for a long time especially over the weekend, Friday to Monday morning before getting treatment because the aid post worker usually goes to his village during weekends. So if the patient is sick on Friday, they have to wait till Monday or find transport to Lenakel hospital*. (primary caregiver, FGD).

There was some notable difference in treatment-seeking behaviours between villages, particularly between the more isolated villages in North and South Tanna and the more easily accessible Middle Bush village. People, from North and South Tanna often reported opting to bypass the village health worker altogether and go directly to the hospital. Reasons given for this practice included the voluntary aid post worker being unavailable, concern regarding severity of the fever, knowledge of time it will take to get to the hospital and ease of physical access (one village described a bad road to their closest aid post). A minority concern (in the case of one village), was the lack of quality of care perceived to be provided by their aid post.

People interviewed in remote areas of North and South Tanna described how land transport was not always easy to find and may be negatively affected by weather, poor roads and a need to seek alternative transport such as a boat. Therefore, opting to go directly to a higher level facility often happened quicker due to knowledge of travel time and preparation time required to organize transport including the possibility of adverse weather conditions. That is, as soon as people perceived a fever to be becoming more serious, preparation for transport to the main hospital on the island commenced

## Discussion

The elimination of malaria in Tafea province within the next few years and a goal for countrywide malaria elimination of parasite transmission in Vanuatu within the next 10-20 years requires a re-orientation of control activity to move away from a programme designed to maintain controlled low-endemic malaria, to one based on effective and more intense surveillance and response that seeks to interrupt endemic transmission and prevent re-establishment [1,32,36,40,41]. The strategy for elimination in Tafae province, Vanuatu currently includes increasing community awareness and participation through dispersal of insecticide treated bed nets, indoor residual house spraying, larviciding and strengthening the health system to increase the availability of malaria diagnostic tests, effective treatment and improved routine reporting [32]. However, sensitive and effective detection of infected individuals also plays a vital role and not only relies on skilled and resourced health services, but also early treatment seeking from community members [42]. Understanding unique treatment-seeking behaviours for fever in populations will assist in identifying the possible barriers to surveillance and response activities that might exist such as reasons for delays in diagnosis and treatment. Furthermore, understanding community behaviour around treatment-seeking, will assist in the sustained community and health system efforts that will be required to prevent the resurgence of malaria following elimination [41].

Even though febrile illnesses (including malaria) in the biomedical sense should require immediate action, studies have rarely shown the use of formal health services as the first resort for diagnoses of malaria [7]. This was also true in Tanna, where perceptions about the cause and appropriate treatment methods to be used can play a large role in the type of healthcare chosen. The practices that Tannese use to differentiate malarial from other fever are based on a number of symptom interpretations that reflect intersections between traditional and contemporary perspectives, socio-cultural dimensions and dynamic relationships between them in an environment with marked cultural and linguistic diversity and as a result, treatment delays are common.

The study showed that people from communities on Tanna recognize malaria as a serious illness of high community priority that has the potential to have a significant impact on the individual and the community, and as previously described, prevention practice was often motivated by risk perception [3]. However, despite the recognition that malaria is a complex disease with potential for devastating results, and assurances that there would be no delay in seeking assistance from the aid post if malaria was suspected, distinguishing malarial fever from other fevers often involved the affected person or caretaker observing symptom progress over a number of days and "trials" with other remedies.

Early treatment-seeking from a health worker typically occurred when it involved a child or a pregnant woman who was sick with fever. Treatment was sort with even more urgency if the community had previous catastrophic experience with malaria, such as death or disability of a child. It was typical for otherwise healthy adults with fever to access health workers after significantly longer time frames due to perception of increased resistance to complications. Participants also tended to consider the aid post for otherwise healthy adults only if the fever did not respond to home remedies or if it was perceived to present unusually, in combination with other symptoms, and for extended periods.

### Sustaining motivation for treatment-seeking of a disappearing disease

#### Increasing prompt treatment seeking for fever within communities

The many difficulties in diagnosing malaria cases and demonstrated difficulties with differentiating it from other illnesses in local disease categories can affect treatment-seeking behaviour and community perceptions of the impact and success of malaria elimination programs. In addition to this, as malaria transmission declines in Tafae province, and the possibility of elimination is increasingly pursued, accurate diagnosis and case identification through passive case detection is even more important [43]. Community awareness and ongoing encouragement by aid post workers for community members to access appropriate treatment quickly for all fevers is imperative particularly where *kastom *medicine and home remedies may be a typical first point of treatment due to various environmental and ideological reasons as demonstrated in this study.

The role of community infrastructure in treatment-seeking for malaria is important and cannot be underestimated [44]. It has been suggested previously that a broader perspective to underlying determinants in the community should be given and that community infrastructure is as important as individual or household determinants [44]. This study showed that in some cases decision to use informal treatments for any member of the family was influenced by health worker availability, access issues and previous experience with biomedical interventions. Trust and reliance on formal health systems and confidence in the health centre are integral to a successful malaria elimination programme [4,14]. Addressing the broader determinants would also assist in strengthening the health system and community trust for current and future public health campaigns. It is also necessary to strengthen overall primary healthcare provisions in order to address other community health and disease priorities within the community, particularly conditions with similar symptom presentations including other febrile and vector-borne illnesses.

#### Recognizing the spectrum of fevers within a community

Recognition that the presentations of certain types of probable malarial signs and symptoms may be uniquely classified as non-malarial diseases by community members and health workers has significant therapeutic and preventive consequences and warrants the attention of any malaria elimination effort [4]. Careful consideration to understand how to manage people who present to the aid post with fever but are diagnosed as not malaria by the health worker, and therefore do not receive treatment for malaria is required. Not receiving treatment, especially when the community member believes they have malaria may result in dissatisfaction with the health services ability to attend to symptoms of fevers. This may lead communities to consider alternative treatments as a first and only point of call. The possibility of more intense symptoms in adults contracting malaria following loss of partial immunity to the disease may also provide confusion within communities that consider complications to malaria to mainly be a childhood issue.

A successful elimination campaign should not address malaria in isolation but recognize it as a part of a wider community of illnesses relevant to indigenous perceptions to ensure ongoing community support. For example, a campaign that concentrates on malaria elimination without addressing the spectrum of fevers and their causes could result in community concern and confusion. Highlighting the importance of prompt treatment for any fever through health promotion campaigns and taking care in ensuring clear communication between health worker and patient at diagnosis is important. Post-elimination on Tanna, trust in the health programme may be eroded if the community perceives fevers that occur amongst children (due to other illnesses) or adults are due to malaria, yet the health staff appear to do nothing. Managing malaria as part of an Integrated Management of Childhood Illness (IMCI) strategy could assist in active engagement of community members in future health system programmes [45]. Low morbidity and incidence of asymptomatic infections weaken the effectiveness of passive case detection in elimination programmes and, therefore, cannot be used in isolation. To avoid onward transmission, the elimination programme will also increasingly need to focus on detecting infections in the general population through active rather than passive case detection [32,43]. For elimination to ultimately succeed, there must be also an effective barrier to reintroduction of the parasite [36]. Therefore, ongoing commitment and motivation from all community members is required, particularly as people cannot completely remain unexposed to the potential for malaria due to other exposure possibilities such as travel and visitors to the island. Ongoing commitment to prompt treatment seeking as well as prevention activities is required. However, motivation for active participation in a malaria elimination programme following high coverage of interventions and attainment of low disease transmission, may be hard to sustain. Programme coordinators need to consider the need to adapt to the potential changing perceptions of communities and how promotion of maintaining vigilance against malaria is incorporated into community awareness campaigns and the activities of frontline health workers.

### Study limitations

The research project involved qualitative research methods. Qualitative research methods are valuable in providing rich descriptions of complex phenomena; illuminating the experience and interpretations of events by actors with widely differing stakes and roles (such as illness and health experiences); and giving voice to those that are rarely heard which is important for understanding appropriate control measures in infectious disease research such as malaria [46]. However, as with the nature of qualitative research, data results are limited in their ability to be generalized to the wider population of Vanuatu.

Discussions about malaria may also have been influenced by social desirability bias due to the fact that the research team was introduced by village leaders as being part of the malaria elimination programme. Apparent contradictions in treatment-seeking behaviours may be explained by respondents seeking to show compliance with Western health practices but in reality often following more traditional health-seeking patterns and practices.

A potential criticism in the selection of study villages is that they were chosen on the basis of their demonstrated leadership diligence in health-related issues and a willingness to co-operate with the malaria elimination programme. However, participants in the focus groups and participatory workshops tended to be from a wide background of ideologies, including those identifying with *Kastom *only or from 'John Frum.'

A further potential limitation of the study is the possible loss of nuances that can occur through the direct translation from *Bislama *to English of the FGD and KII recordings by the local research officers. Although the study team includued researchers' native to Tanna, they were not fluent in all the seven local dialects present on the island. Therefore research was conducted in either English or the national language of *Bislama*. For participants in the FGDs who did not speak English, *Bislama *or a known local dialect to the researchers, translation was carried out by other participants.

## Conclusion

Early diagnosis and prompt case management is an important component of the malaria elimination strategy in Tafea Province, Vanuatu. However, its success requires interactions between the community and its leaders, malaria workers and health providers. This study explored community perceptions and understandings of malaria, its symptoms, causation and how these factors influence treatment-seeking behaviours. As malaria becomes more uncommon, motivating communities to present for early treatment for fever will become increasingly difficult and therefore utilizing and identifying unique motivators for communities to seek early diagnosis and treatment is important particularly as other health conditions that cause fevers become increasingly more common and dependent upon robust understanding of community perceptions of disease. Eliminating malaria without reducing the incidence of fever from other causes may also leave communities questioning the real impact of the programme in improving their overall health. The content of behaviour change communication targeted at illness recognition and prompt treatment should incorporate appropriately the traditional beliefs, so that the community is aware that their perceptions are acknowledged and key messages are meaningful within prevailing constructions of health and disease.

## Competing interests

The authors declare that they have no competing interests.

## Authors' contributions

JA, HT, LF, GT, IR, MW & AV participated in the conception of study design. Training of field researchers in qualitative methods and logistics was carried out by LF, JA & HT. The field research activities were supported by AT, JA & HT. Data analysis and manuscript drafting was carried out by AT and MS with support and contributions from AV, LF, MW & JA. All authors have read and approved the final manuscript.
